# Supplementary material for: Structural insights into the regulation, ligand recognition, and oligomerization of bacterial STING
Source: Nat Commun. 2023 Dec 21;14:8519. doi: 10.1038/s41467-023-44052-x (PMC10739871; doi:10.1038/s41467-023-44052-x)
Supplement: Supplementary file 1 — Supplementary Information [file 41467_2023_44052_MOESM1_ESM.pdf]

Supplementary Tables

Supplementary Table 1. Data collection and refinement statistics of *Ri*STING crystal.

	***Ri*STING (PDB 8HYN)**	***Ri*STING_cGG (PDB 8HY9)**
**Data collection**		
Space group	$P2_12_12_1$	$C2$
Cell dimensions		
a, *b*, *c* (Å)	53.27, 60.12, 118.82	99.51, 40.99, 36.20
α, β, γ (°)	90, 90, 90	90, 105, 90
Resolution (Å)	30−2.10 (2.18−2.10)	30−1.46 (1.51−1.46)
Unique reflections	22890 (2235)	24248 (2302)
Completeness (%)	98.3 (97.4)	99.3 (95.0)
Redundancy	7.1 (7.1)	6.2 (4.7)
R_{merge} (%)	4.2 (30.1)	5.4 (48.9)
R_{pim} (%)	1.7 (12.1)	2.3 (24.3)
I/σ *(I)*	43.1 (5.8)	30.2 (2.9)
$CC_{1/2}$	(0.958)	0.996 (0.834)
**Refinement**		
No. reflections	22787 (2135)	24110 (2088)
R_{work} / R_{free}	19.3 / 24.3	18.7 / 20.6
No. atoms		
Protein	2553	1286
Ligand/ion	26	47
Water	360	124
B-factors		
Protein	34.4	18.1
Ligand/ion	47.2	10.7
Water	43.6	26.6
R.m.s. deviations		
Bond lengths (Å)	0.014	0.015
Bond angles (°)	1.65	1.59

*Values in parentheses are for highest-resolution shell.

Supplementary Table 2. SWAXS data collection parameters and processing details.

Sample name	apo *Ri*STING dimer	*Ri*STING dimer + cGG
(a) Sample details		
Organism	*Riemerella anatipestifer* Yb2	
GenBank ID (residues in construct)	AKQ40609.1 (158-316)	
Ligand – PubChem CID	-	135440063
Calculated molecular weight (Da)	37092.04	37092.04 (*Ri*STING) + 690.4 (cGG) = 37782.44
SEC column	Agilent Bio SEC-3 LC Column (pore size 300 Å)	
Number of frames used for data analysis	5	
Injected volume (µL)	100	
Loading concentration (mg/mL)	10	
Flow rate (mL/min)	0.2 and 0.3	
SEC buffer	50 mM tris; 200 mM NaCl; 1 mM TCEP; 5% Glycerol	
(b) SAXS data collection parameters		
Instrument	TPS 13A BioSWAXS beamline of the National Synchrotron Radiation Research Center	
Wavelength (Å)	0.8265	
q range (Å^{-1})	0.005 – 0.4 (SAXS); 0.2 – 1.7 (WAXS)	
Sample-to-detector distance (m)	2.8	
Exposure time	2 sec/frame	
Temperature (°C)	10	
Detector(s)	Eiger X 9M (SAXS) and X 1M (WAXS) detectors both in vacuum	
Flux (photons/s)	~1x10^{12}	
Beam size (µm)	300(H)x225(V)	
Sample configuration	2.0 mm diameter quartz capillary	
Absolute scaling method	Scaling to absolute water scattering intensity	
Normalization	Accumulated monitor counts of the incident X-ray flux over the sample measuring time.	
(c) Structural parameters		
R_g (Å) [from p(r)]	22.7±0.1	22.3±0.1
R_g (Å) [from Guinier]	22.9±0.1	22.0±0.1

D_{max} (Å)	79.7	71.9
MW determined from size & shape (kDa)	31.2	36.7
MW determined from V_c (kDa)	31.5	36.5
(d) Software employed		
SAXS data reduction and data processing	TPS 13A SWAXS Data Reduction Kit (Ver. 3.6)<br>PRIMUS (ATSAS 3.1.3)	
Bead modelling	GASBOR (ATSAS 3.1.3)	
Computation of model intensities	CRYSOL (ATSAS 3.1.3)	

Supplementary Table 3. The list of *Lr*STING-like proteins.

GenBank or NCBI accession	Species	Protein size (a.a.)
MCC6655243.1	*Flavobacteriales bacterium*	328
WP_160318893.1	*Arthrobacter sp.* ERGS1:01	328
RYE18178.1	*Sphingobacteriaceae bacterium*	328
OJW70287.1	*Spirosoma sp.* 48-14	328
WP_150091374.1	*Adhaeribacter rhizoryzae*	329
WP_091164037.1	*Mucilaginibacter gossypii*	322
PKQ44211.1	*Confluentibacter flavum*	321
MBX3059614.1	*Anaerolineae bacterium*	322
WP_129463058.1	*Flavobacterium piscinae*	321
WP_089804445.1	*Chitinophaga sp.* YR627	324
WP_169533664.1	*Hymenobacter polaris*	323
WP_158096597.1	*Pseudoflavonifractor sp.* An187	332
WP_243512984.1	*Hymenobacter monticola*	328
VWX61582.1	*Burkholderiales bacterium* 8X	335
WP_004847972.1	*Ruminococcus torques*	323
MBA3963795.1	*Chthoniobacterales bacterium*	325
MBF8293921.1	*Bacteroidetes bacterium*	345

Supplementary Table 4. Data collection and refinement statistics of *Lr*STING crystal.

	***Lr*STING_cGG (PDB 8HWI)**
**Data collection**	
Space group	$P2_12_12_1$
Cell dimensions	
a, *b*, *c* (Å)	67.99, 72.49, 231.07
α, β, γ (°)	90, 90, 90
Resolution (Å)	30−2.72 (2.82−2.72)
Unique reflections	30140 (2870)
Completeness (%)	96.5 (93.5)
Redundancy	10.3 (8.3)
R_{merge} (%)	9.9 (152.6)
R_{pim} (%)	3.2 (53.1)
I/σ *(I)*	23.3 (1.3)
$CC_{1/2}$	(0.574)
**Refinement**	
No. reflections	26830 (1318)
R_{work} / R_{free}	19.8 / 25.1
No. atoms	
Protein	8812
Ligand/ion	138
Water	242
B-factors	
Protein	66.29
Ligand/ion	26.65
Water	49.22
R.m.s. deviations	
Bond lengths (Å)	0.005
Bond angles (°)	0.89

Supplementary Table 5. Data collection and refinement statistics of *El*STING crystals.

	*El*STING (PDB 8HY8)	*El*STING_cAA (PDB 8HWJ)
**Data collection**		
Space group	$P6_522$	*C*2
Cell dimensions		
a, *b*, *c* (Å)	56.98, 56.98, 187.55	157.17, 52.71, 88.93
α, β, γ (°)	90, 90, 120	90, 114.88, 90
Resolution (Å)	30−2.56 (2.65−2.56)	30−2.55 (2.64−2.55)
Unique reflections	6344 (572)	20886 (1948)
Completeness (%)	99.2 (95.0)	96.5 (90.4)
Redundancy	18.8 (8.9)	6.3 (5.6)
R_{merge} (%)	17.9 (109.0)	12.1 (57.8)
R_{pim} (%)	4.0 (35.8)	5.2 (26.2)
I/σ *(I)*	20.5 (1.4)	14.1 (2.3)
$CC_{1/2}$	0.991 (0.696)	0.993 (0.806)
**Refinement**		
No. reflections	5899 (319)	19580 (1302)
R_{work} / R_{free}	21.4 / 24.3	19.6 / 22.6
No. atoms		
Protein	1235	5151
Ligand/ion	-	88
Water	41	172
B-factors		
Protein	43.5	48.9
Ligand/ion	-	25.3
Water	36.0	41.2
R.m.s. deviations		
Bond lengths (Å)	0.014	0.013
Bond angles (°)	1.77	1.83

*Values in parentheses are for highest-resolution shell.

Supplementary Table 6. The sequences of the codon-optimized genes used in this study.

Riemerella anatipestifer **TIR-STING$^{wild\text{-}type}$ (*Ri*TIR-STING^{WT})**
ATGCGCAAGAAACGCATTTTTATTGGCAGCAGCAGCGAAGAACTGGATCTGGCGAGCGCGGCG<br>AAAAGCATTCTGGAACTGGAAAAAGAATTTGAAGTGACCATTTGGAACGAAGATGTGTGGGAA<br>AAAGCGGTGTTTCGCCTGAACAACAGCTATCTGAACGATCTGATTCGCGCGACCCTGCAGTTTG<br>ATTTTGGCATTCTGATTGGCACCAAAGACGATAAAGTGGTGTATCGCGGCAACGAAGAACTGCA<br>GCCGCGCGATAACATTCTGTTTGAACTGGGCCTGTTTATTGGCCGCCTGGGCCTGAACAACTGC<br>GCGTTTCTGATTGATAAAGATATCAAACTGCTGAGCGACATTAAGGGCATTAGCCTGGCGCGCTT<br>TAACCGCGGCGATAGCAGCAGCTTTACCAAAGCGATTACCCAGGTGAAAGATCTGTTAAAAAC<br>CAGGTGGATAGCGGCATTAACTTCTTTCCGAGCAGCACCCTGGCGGCGGTGTATTATGAAAACT<br>TTGTGAAACCGACCTGCCTGCATATTATTCAGAACGGCGGCATTCAGGATGATGATGGCACCAA<br>ATATGAAAACAGCACCATTAAGATCATTATCCCGCAGAAACTGACCACCGATGTGAACAGCCAG<br>TTTCAGACCCTGAAGAAAAGCTTTCAGACCAAGAAACTGACCTTTGATTATCTGGGCCGCCCGC<br>GCAACATTGATGTTGAAACCCTGATTCAGGATGGCAAACTGTATGTGATTGATTTTCCGACCGTG<br>CTGAGCGGCATTAACTATGCGATTAGCAACCTGCTGCCGAACGATTTTAACAGCATGAGCGATG<br>ATTATGAACTGATTCTGAACCGCGAATTTGATCGCTTTATTTACACCCTGAACAAACTGGCGCTG<br>CGCGATGGCTATAACAACCTGATTACCGTGATTAACGAAAAGGACATCAAACTCGAGCACCACC<br>ACCACCACCACTGA
Riemerella anatipestifer **TIR-STING^{G302E} (*Ri*TIR-STING^{G302E})**
ATGCGCAAGAAACGCATTTTTATTGGCAGCAGCAGCGAAGAACTGGATCTGGCGAGCGCGGCG<br>AAAAGCATTCTGGAACTGGAAAAAGAATTTGAAGTGACCATTTGGAACGAAGATGTGTGGGAA<br>AAAGCGGTGTTTCGCCTGAACAACAGCTATCTGAACGATCTGATTCGCGCGACCCTGCAGTTTG<br>ATTTTGGCATTCTGATTGGCACCAAAGACGATAAAGTGGTGTATCGCGGCAACGAAGAACTGCA<br>GCCGCGCGATAACATTCTGTTTGAACTGGGCCTGTTTATTGGCCGCCTGGGCCTGAACAACTGC<br>GCGTTTCTGATTGATAAAGATATCAAACTGCTGAGCGACATTAAGGGCATTAGCCTGGCGCGCTT<br>TAACCGCGGCGATAGCAGCAGCTTTACCAAAGCGATTACCCAGGTGAAAGATCTGTTAAAAAC<br>CAGGTGGATAGCGGCATTAACTTCTTTCCGAGCAGCACCCTGGCGGCGGTGTATTATGAAAACT<br>TTGTGAAACCGACCTGCCTGCATATTATTCAGAACGGCGGCATTCAGGATGATGATGGCACCAA<br>ATATGAAAACAGCACCATTAAGATCATTATCCCGCAGAAACTGACCACCGATGTGAACAGCCAG<br>TTTCAGACCCTGAAGAAAAGCTTTCAGACCAAGAAACTGACCTTTGATTATCTGGGCCGCCCGC<br>GCAACATTGATGTTGAAACCCTGATTCAGGATGGCAAACTGTATGTGATTGATTTTCCGACCGTG<br>CTGAGCGGCATTAACTATGCGATTAGCAACCTGCTGCCGAACGATTTTAACAGCATGAGCGATG<br>ATTATGAACTGATTCTGAACCGCGAATTTGATCGCTTTATTTACACCCTGAACAAACTGGCGCTG<br>CGCGATGAATATAACAACCTGATTACCGTGATTAACGAAAAGGACATCAAACTCGAGCACCACC<br>ACCACCACCACTGA
Riemerella anatipestifer **TIR-STING$^{N270E/N273E/Y292E}$ (*Ri*TIR-STING$^{N270E/N273E/Y292E}$)**
ATGCGCAAGAAACGCATTTTTATTGGCAGCAGCAGCGAAGAACTGGATCTGGCGAGCGCGGCG<br>AAAAGCATTCTGGAACTGGAAAAAGAATTTGAAGTGACCATTTGGAACGAAGATGTGTGGGAA

AAAGCGGTGTTTCGCCTGAACAACAGCTATCTGAACGATCTGATTCGCGCGACCCTGCAGTTTG<br>ATTTTGGCATTCTGATTGGCACCAAAGACGATAAAGTGGTGTATCGCGGCAACGAAGAACTGCA<br>GCCGCGCGATAACATTCTGTTTGAACTGGGCCTGTTTATTGGCCGCCTGGGCCTGAACAACTGC<br>GCGTTTCTGATTGATAAAGATATCAAACTGCTGAGCGACATTAAGGGCATTAGCCTGGCGCGCTT<br>TAACCGCGGCGATAGCAGCAGCTTTACCAAAGCGATTACCCAGGTGAAAGATCTGTTTAAAAAC<br>CAGGTGGATAGCGGCATTAACTTCTTTCCGAGCAGCACCCTGGCGGCGGTGTATTATGAAAACT<br>TTGTGAAACCGACCTGCCTGCATATTATTCAGAACGGCGGCATTCAGGATGATGATGGCACCAA<br>ATATGAAAACAGCACCATTAAGATCATTATCCCGCAGAAACTGACCACCGATGTGAACAGCCAG<br>TTTCAGACCCTGAAGAAAAGCTTTCAGACCAAGAAACTGACCTTTGATTATCTGGGCCGCCCGC<br>GCAACATTGATGTTGAAACCCTGATTCAGGATGGCAAACTGTATGTGATTGATTTTCCGACCGTG<br>CTGAGCGGCATTAACTATGCGATTAGCAACCTGCTGCCGGAAGATTTTGAAAGCATGAGCGATG<br>ATTATGAACTGATTCTGAACCGCGAATTTGATCGCTTTATTGAAACCCTGAACAAACTGGCGCTG<br>CGCGATGGCTATAACAACCTGATTACCGTGATTAACGAAAAAGATATCAAACTCGAGCACCACC<br>ACCACCACCACTGA
***Larkinella arboricola* TIR-STING$^{wild-type}$ (*Lr*TIR-STING^{WT})**
ATGAGCAAGAAAAAAGAGACCGTCAACAAGGCCATGAAACCGACCATCTTCATCGCAAGCAG<br>CGGTAAAAGCAGCGATATTGCAGAGGCGATCAAACTGAACCTGGACAAAGAGGCGGAAGTTG<br>ATATTTGGACCGAGAACATCTTTCAGCAGAACGAAGGCACCCTGGAAACCCTGATGAATCGCG<br>CGAGCTATTACGACTTCTTCATTGGCGTTTTTGCGGCAGACGATACCGCGATCATCAAGAAGAA<br>GAAGAAGGACGTCACCCGCGATAACGTCATCTTCGAATTTGGCCTGTTTCTGGGGCGTATTGGT<br>CTGGATCGTACGTTCTTCGTCCTGGAAGAAGGCATCGACCTGTTTAACGACTGGAACGGCATTA<br>CCACCAGTACCTTTACCCGTCGCGATAATCTGACCTCTGCACTGGGCGCAAGCTGTATTCGTATT<br>AAAGAACGCATGAAGGTTGCGGAAGAACTGTTTAACTATACCGTCCTGCCGAGTACCAGCCTG<br>GCGGTCGGCTATTATTACAACTTCCTGCGCGAAATCCTGGAAGCGTTTAACAACCAGAAAAGCA<br>TCCAGATCATTCTGGAACGCGATCGTACCGGTAAACCGACCAAAACCATCGACTACGAAATCAA<br>GAAGCCGTATCCGACCATCGAAATTCGCGTTCCGCAGAATCTGGCGAGCCTGAAAAAAGAGGT<br>CCTGACCTGGAATACCAGCGAATACAAGCAGATCTTCATTAACGCGGCGAGCCGTACCTATCCG<br>TTTTTCCTGCAGGGCGAATTTAAAGAAGATCAGATCCTGAGCATCTTCGATATTCCGACCACCCT<br>GTACGCAAGCTATCTGACCATTAAAGAACTGTTCACCGACTCCTTCCTGAAAACCCAGAACAAC<br>GAGCGCAAGCTGATCAACAAAGAAATTCGCAACTTCGAACGTACCCTGAGCAAACTGATCGAC<br>GATACCATCGAAGAAAAATTCTACAAGTTCACCATCTACCTCGAGCACCACCACCACCACCACT<br>GA
***Larkinella arboricola* TIR-STING$^{lid \rightarrow loop}$ (*Lr*TIR-STING $^{lid \rightarrow loop}$)**
ATGAGCAAGAAAAAAGAGACCGTCAACAAGGCCATGAAACCGACCATCTTCATCGCAAGCAG<br>CGGTAAAAGCAGCGATATTGCAGAGGCGATCAAACTGAACCTGGACAAAGAGGCGGAAGTTG<br>ATATTTGGACCGAGAACATCTTTCAGCAGAACGAAGGCACCCTGGAAACCCTGATGAATCGCG<br>CGAGCTATTACGACTTCTTCATTGGCGTTTTTGCGGCAGACGATACCGCGATCATCAAGAAGAA<br>GAAGAAGGACGTCACCCGCGATAACGTCATCTTCGAATTTGGCCTGTTTCTGGGGCGTATTGGT

CTGGATCGTACGTTCTTCGTCCTGGAAGAAGGCATCGACCTGTTTAACGACTGGAACGGCATTA<br>CCACCAGTACCTTTACCCGTCGCGATAATCTGACCTCTGCACTGGGCGCAAGCTGTATTCGTATT<br>AAAGAACGCATGAAGGTTGCGGAAGAACTGTTTAACTATACCGTCCTGCCGAGTACCAGCCTG<br>GCGGTCGGCTATTATTACAACTTCCTGCGCGAAATCCTGGAAGCGTTTAACAACCAGAAAGGTA<br>GTGGTGGCGGCGGTTCCGGCGGCGGTGGTAGTGGCGGCGGCGGTAGCGGCGGCGGTAGTATCA<br>AGAAGCCGTATCCGACCATCGAAATTCGCGTTCCGCAGAATCTGGCGAGCCTGAAAAAAGAGG<br>TCCTGACCTGGAATACCAGCGAATACAAGCAGATCTTCATTAACGCGGCGAGCCGTACCTATCC<br>GTTTTTCCTGCAGGGCGAATTTAAAGAAGATCAGATCCTGAGCATCTTCGATATTCCGACCACCC<br>TGTACGCAAGCTATCTGACCATTAAAGAACTGTTCACCGACTCCTTCCTGAAAACCCAGAACAA<br>CGAGCGCAAGCTGATCAACAAAGAAATTCGCAACTTCGAACGTACCCTGAGCAAACTGATCGA<br>CGATACCATCGAAGAAAAATTCTACAAGTTCACCATCTACCTCGAGCACCACCACCACCACCAC<br>TGA
Epilithonimonas lactis **TIR-STING$^{\text{wild-type}}$ (*El*TIR-STING^{WT})**
ATGCGTACCCGTATTTTCATTGGCAGCAGCAAAGAGGGCCTGAAAGAAGCGAACTACGTCAAA<br>AGCCGCCTGGAAAAAGCGAACTTCGAGGTCTTCATCTGGAACGACGACATCTTCAAACCGAAC<br>AAAAACACCCTGGAAACCCTGCTGAACGTTGCGAGCCTGTTTGATTTCGGCATCATGATCGCGA<br>CCAAAGACGATTTTACCGCGAGTCGCGACGATATCTTTGAAACCGTTCGCGACAACGTCGTCTT<br>CGAATTTGGCCTGTTTCTGGGTCGTCTGGGCGAAAATCGCGCATTTGCACTGCAAGAAAACGGC<br>GCAAAACTGCCGTCTGATCTGCTGGGTATCACCATCCCGAAATTCGAGAAAACCGACGACTACT<br>TCAGCAACTACAACCTGAACACCGAGATCGACAACATCATCAAAATCATCAACGAGAAAATCA<br>GCCTGGGCGAACTGGGTCTGCTGCCGAGTACCGTTCTGGCGATTGGCTATTACGAGAACTTCGT<br>CAGTACCGTTTGCGACGCACTGCATAGTCTGCCGACCATTAAACTGAACGGCATCGAGTACAAA<br>GACTTCGTCTTCAACATCATCATCCCGAACGATCTGGACGCAGATATTAAACGCCGCGCGCAGA<br>TCTACTTCAAAAAGATGGACATCCACGAAGTCAAAATCGATACCAACGGCCGTAGTTTTCCGCT<br>GTATCTGCAGATCGACGAAGAAAACAGCGGCGACGTTGCAGTTCTGTACGATATGCCGACCACC<br>CTGGGCGGTATTGATAAAGCCATCGAGATGTACATGAAAAAGGCCACATTGGTAAAACCAGCC<br>AGCAGCAACTGCTGGAAGAACGCGAACTGCGCAACTTCAAAACCACCCTGATCAACCTGATCA<br>ACAACAACAGCTTCACCAAAACCTTCGTCAAAGTCATCGAAGAACTCGAGCACCACCACCACC<br>ACCACTGA
Epilithonimonas lactis **TIR-STING$^{\text{D175R, S179R}}$ (*El*TIR-STING$^{\text{D175R, S179R}}$)**
ATGCGCACCCGCATTTTTATTGGCAGCAGCAAAGAAGGCCTGAAAGAAGCGAACTATGTGAAA<br>AGCCGCCTGGAAAAAGCGAACTTTGAAGTGTTCATTTGGAACGACGATATCTTCAAGCCGAAC<br>AAAAACACCCTGGAAACCCTGCTGAACGTGGCGAGCCTGTTTGATTTTGGCATTATGATTGCGA<br>CCAAAGATGATTTTACCGCGAGCCGCGATGATATTTTTGAAACCGTGCGCGATAACGTGGTGTTT<br>GAATTTGGCCTGTTTCTGGGCCGTCTGGGCGAAAACCGCGCGTTTGCGCTGCAAGAAAACGGT<br>GCGAAACTGCCGAGCGATCTGCTGGGCATTACCATTCCGAAATTCGAGAAAACCGACGATTACT<br>TTAGCAACTACAACCTGAACACCGAAATTGACAACATCATCAAGATCATCAACGAGAAAATTAG<br>CCTGGGCGAACTGGGTCTGCTGCCGAGTACCGTGCTGGCGATTGGTTATTATGAAAACTTTGTG

AGCACCGTTTGCCGCGCACTGCATCGCCTGCCGACCATTAAACTGAACGGCATCGAATACAAGG<br>ATTTCGTGTTCAACATCATCATTCCGAACGATCTGGATGCGGATATTAAACGCCGCGCGCAGATT<br>TATTTCAAGAAAATGGACATCCACGAGGTGAAAATTGACACCAACGGCCGCAGCTTTCCGCTGT<br>ATCTGCAGATTGATGAAGAAAACAGCGGCGATGTGGCGGTGCTGTATGATATGCCGACCACCCT<br>GGGTGGCATTGATAAAGCGATTGAGATGTATATGAAGAAAGGCCATATCGGCAAAACCAGCCAG<br>CAGCAGCTGCTGGAAGAACGCGAACTGCGCAACTTTAAAACCACCCTGATTAACCTGATCAAC<br>AACAACAGCTTCACCAAGACCTTTGTGAAAGTGATTGAAGAACTCGAGCACCACCACCACCAC<br>CACTGA
**CdaS^{L44F} from *Bacillus subtilis* strain 168**
ATGGGGGGTTCTCATCATCATCATCATCACGGTTCTGGCGGTATGAAAGCGATGCGTTACGAACA<br>GATCAGCGAGAACGCGTTCAAAGGCAAAATCCAGGTCTACCTGGAACAAATCCTGGGCGACGC<br>AAGTCTGATCCTGAAAACCCTGCACGAAAAAGACCAGTGCCTGTTTTGCGAACTGGACGATCT<br>GGGTCACGTCTTTCAGGATATGCAGGGTATCGCGAGCAGCTTTTACCTGCAGAGCTATATCGAA<br>GAATTTACCCCGGCGTTTATTGAACTGGCAAAAGCGATCAAAGCGCTGAGCGAACATAAACAC<br>GGCGCGCTGATTGTTATTGAACGCGCAGATCCGGTTGAACGCTTCATCCAGAAAGGCACCAGCC<br>TGCACGCAGAAATTAGTAGCAGCCTGATCGAAAGCATCTTCTTTCCGGGTAATCCGCTGCACGA<br>CGGCGCACTGCTGGTACGCGAAAATAAACTGGTTAGCGCGGCAAACGTTCTGCCGCTGACCAC<br>CAAAGAAGTCGATATTCATCTGGGCACCCGCCATCGCGCTGCTCTGGGTATGTCTGGTTATACCG<br>ACGCACTGGTTCTGGTTGTCAGCGAAGAAACCGGCAAAATGAGCTTTGCGAAAGACGGCGTTC<br>TGTACCCGCTGATTAGTCCGCGTACCTAA

Supplementary Figures

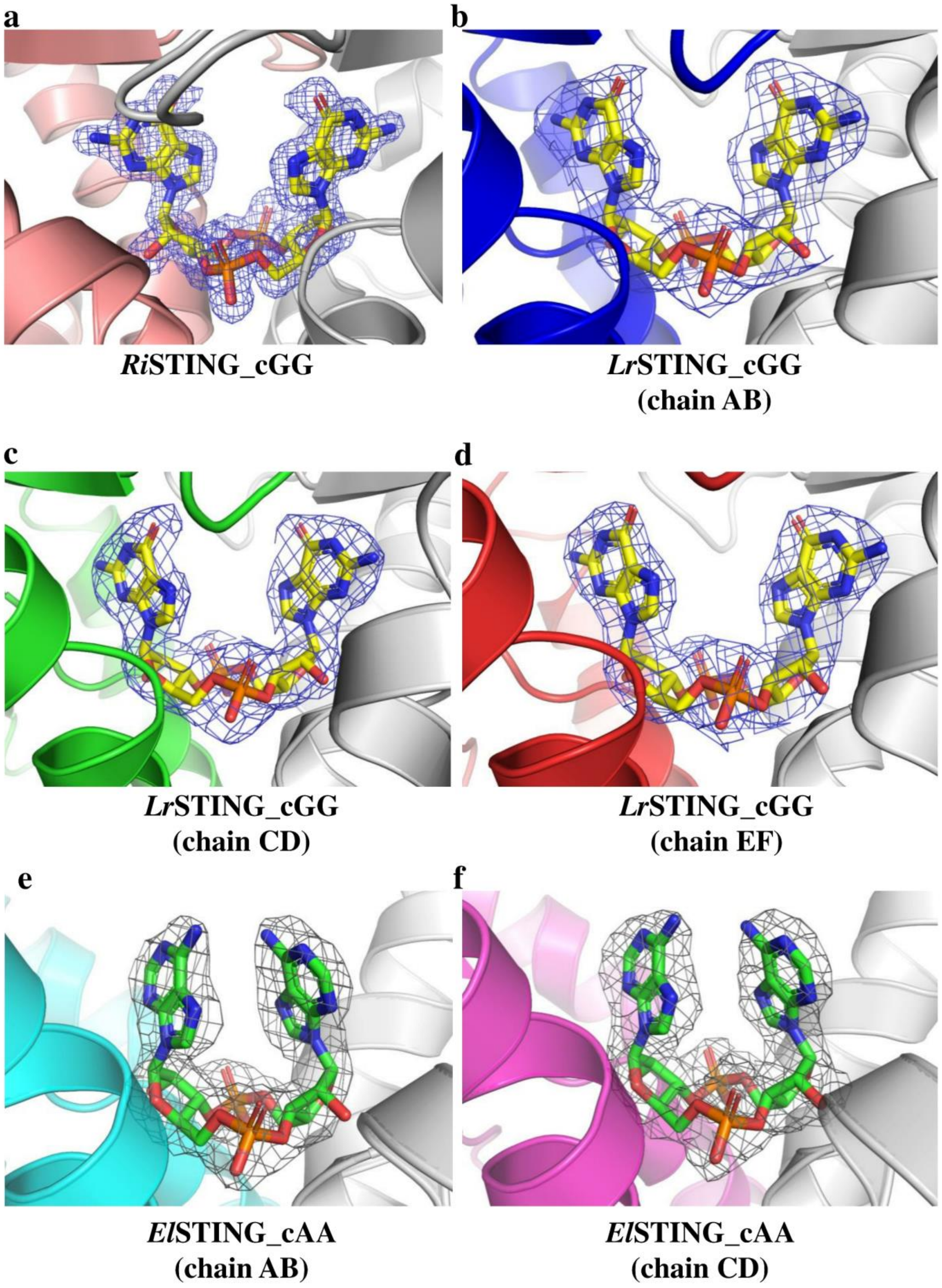

Supplementary Figure 1. Omitted electron-density map (2*F*o−*F*c) of the bound cGG in (a) *Ri*STING, (b–d) *Lr*STING, and the bound cAA in (e, f) *El*STING, contoured at the 1σ level.

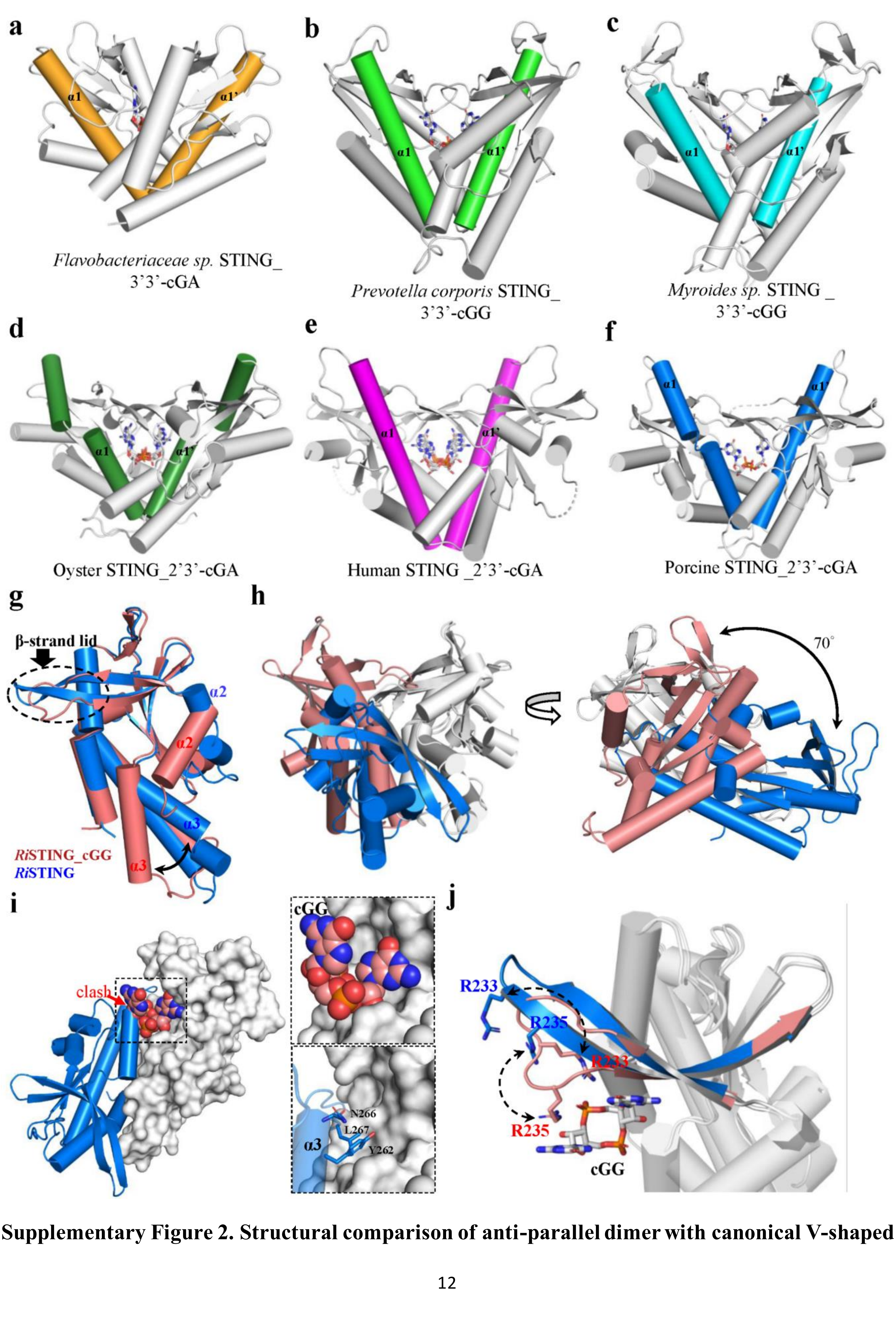

Supplementary Figure 2. Structural comparison of anti-parallel dimer with canonical V-shaped

dimer formed by different bacterial and eukaryotic STINGs.

(a–f) All cyclic di-nucleotide (CDN)-bound STINGs form V-shaped dimeric architectures. (a) *Fs*STING_3'3'-cGA (PDB: 6WT4), (b) *Pc*STING_3'3'-cGG (PDB: 7EBD), (c) *My*STING_3'3'-cGG (PDB: 7EBL), (d) oyster STING_2'3'-cGA (PDB: 6WT7), (e) human STING_2'3'-cGA (PDB: 4KSY) and (f) porcine STING_2'3'-cGA (PDB: 6A06). (g) Superimposition of the protomer of ligand-free, anti-parallel *Ri*STING (marine) with cGG-bound *Ri*STING (salmon). The structural differences between β-strand lids, α2 and α3 helices are indicated. (h) Structural alignment of the anti-parallel *Ri*STING dimer with cGG-bound V-shaped *Ri*STING dimer. With one of the two protomers superimposed, the other protomer undergo a rigid-body rotation of ~70° in the presence/absence of cGG. (i) Left, superimposition of anti-parallel *Ri*STING dimer with cGG-bound *Ri*STING dimer. The protein model of cGG-bound *Ri*STING is neglected for simplicity. The bound cGG (salmon spheres) will make steric clashes with the α3 helix of anti-parallel *Ri*STING protomer (red arrow). Top right, the enlarged view of the ligand-binding pocket of *Ri*STING protomer in complex with cGG. Bottom right, the terminus of α3 helix of one anti-parallel *Ri*STING protomer (residues 262-269) make extensive contacts with the half of the ligand-binding pocket of the other protomer. The sidechains of Y262, N266 and L267 of anti-parallel *Ri*STING, which extend into the ligand-binding pocket, will make steric clashes with cGG. (j) Structural comparison of the β-strand lid of anti-parallel *Ri*STING with that of cGG-bound *Ri*STING. The cGG and the recognition residues R233 and R235 are shown in sticks. The structural movements before and after cGG binding are indicated by black dashed arrows.

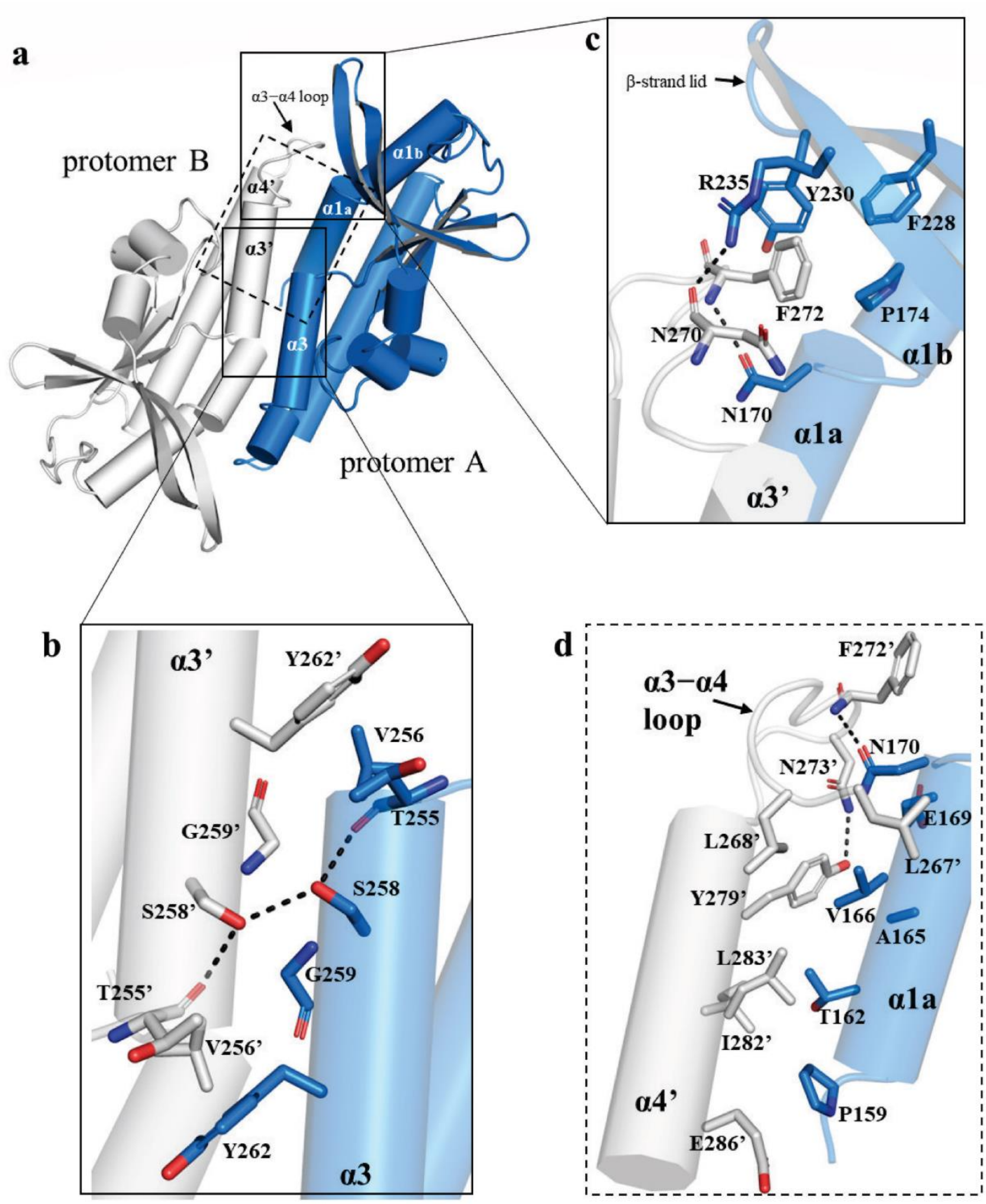

Supplementary Figure 3. Detailed analysis of the dimerization interface of anti-parallel *Ri*STING.

(a) The overview of the dimerization interfaces (indicated by rectangles) of anti-parallel *Ri*STING dimer. (b–d) The detailed view of the interface between (b) two α3 helices of each protomer, (c) α4 helix from one protomer and α1 helix from the other, (d) α3-α4 loop from one protomer and α1 helix and β-stand lid from the other. The hydrogen bonds are indicated by black dashed lines. The residues involved in dimerization are shown in sticks and indicated.

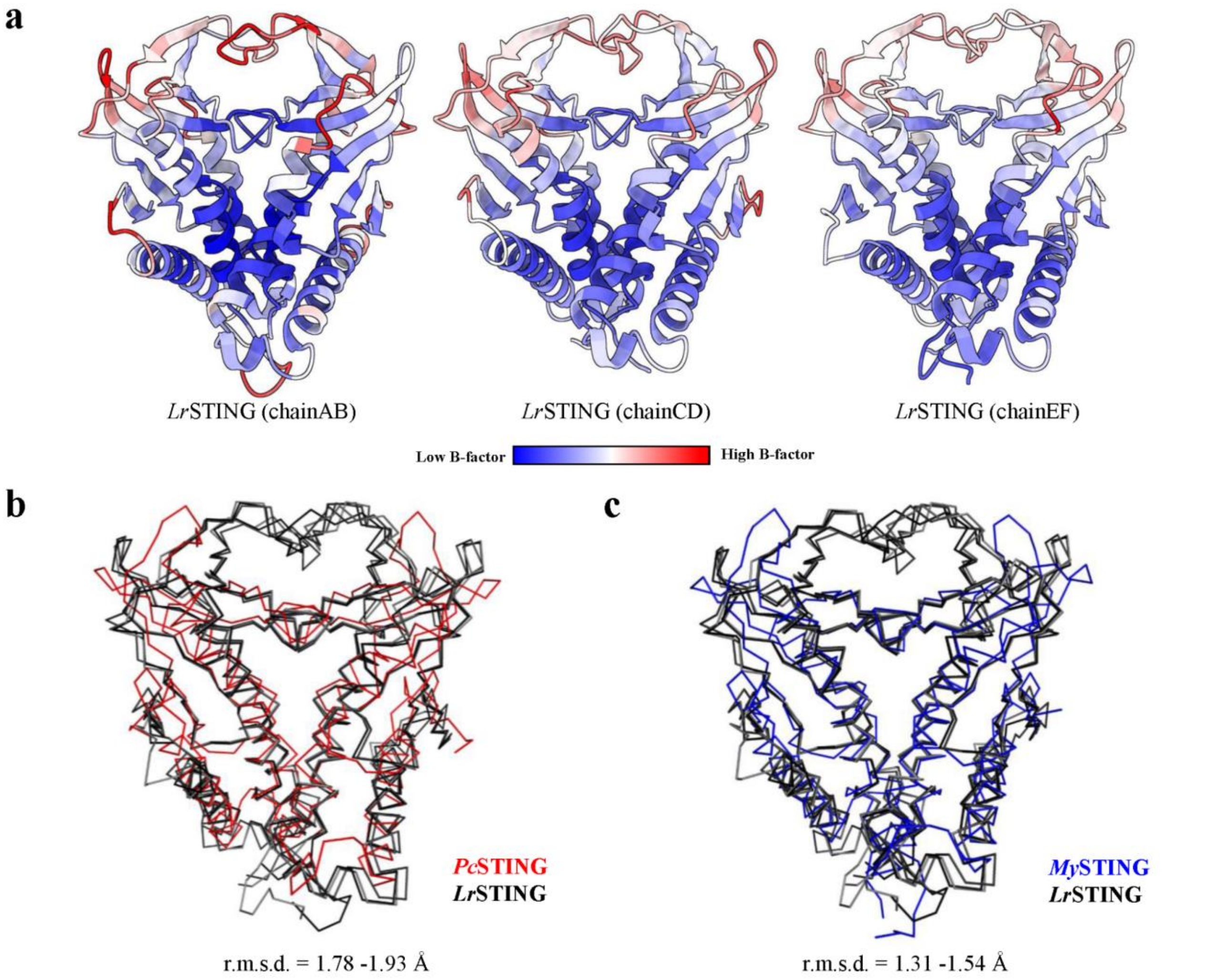

Supplementary Figure 4. Structural comparative analysis of *Lr*STING.

(a) B-factor coloring of the three *Lr*STING dimers in the same asymmetric unit. Higher B-factor values are represented as red, whereas lower B-factor values are represented as blue.

(b, c) Superimposition of (b) *Pc*STING (red) and (c) *My*STING (blue) with the three *Lr*STING dimers (black, grey, and light grey).

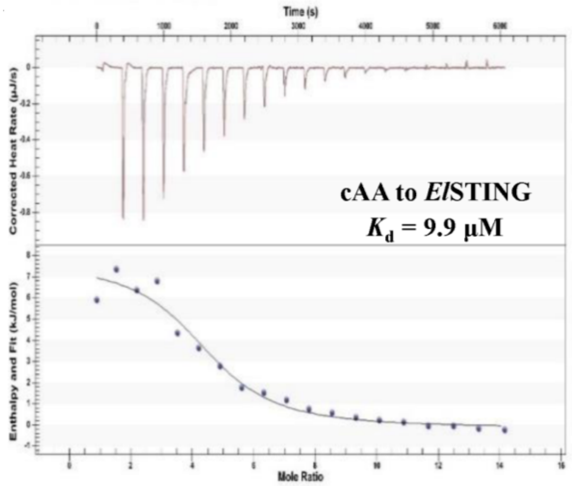

Supplementary Figure 5. Isothermal titration calorimetry (ITC) analysis of cAA binding to *El*STING.

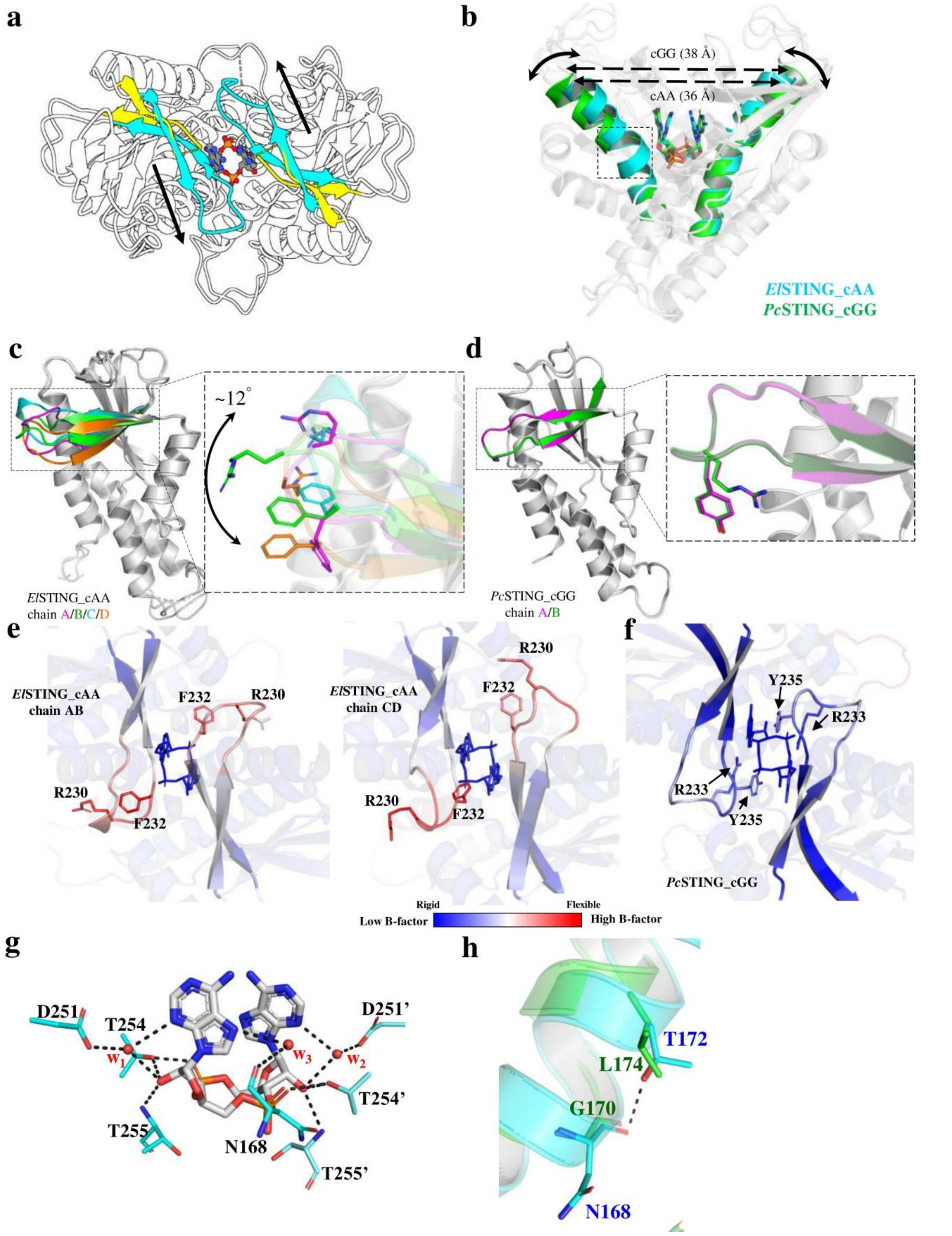

Supplementary Figure 6. Structural comparison of cAA-bound bacterial STING with cGG-bound bacterial STING.

(a) Superimposition of *El*STING (yellow) with *El*STING_cAA complex (cyan). The β-strand lids of

them are highlighted. Binding of cAA stabilizes the formation and induces the closure of β-strand lid of *El*STING (black arrows).

(b) Structural comparison of α1 stem helices between *Pc*STING_cGG (green, PDB: 7EBD) and *El*STING_cAA (cyan) complex. The conformational changes between them are indicated by black arrows.

(c, d) Left, superimposition of protomers of (c) *El*STING_cAA and (d) *Pc*STING_cGG. The β-strand lids of them are colored accordingly. Right, the enlarged view of the ligand recognition residues in β-strand lids. The ligand recognition residue R230 and F232 in Rx(Y/F) motif of *El*STING showed large conformational changes between the four protomers (~12° rotation) in contrast to the nearly identical position of R233 and Y235 of *Pc*STING.

(e, f) B-factor coloring of the β-strand lids of (e) *El*STING_cAA composed of chain A, B (left) and chain C, D (right) and (f) *Pc*STING_cGG. Higher B-factor values are represented as red, whereas lower B-factor values are represented as blue.

(g) Recognition of the adenine bases and 3'3'-phosphodiester linkage of cAA by *El*STING. Water molecules are shown in red spheres. The residues involved in hydrogen bonding (black dashed lines) are shown in sticks.

(h) The enlarged view of the kink at the middle of α1 helices of *Pc*STING (green) and *El*STING (cyan). The sidechain of T172 forms a hydrogen bond with the backbone amide oxygen of N168 of *El*STING; however, the L174 of *Pc*STING at the equivalent position does not.

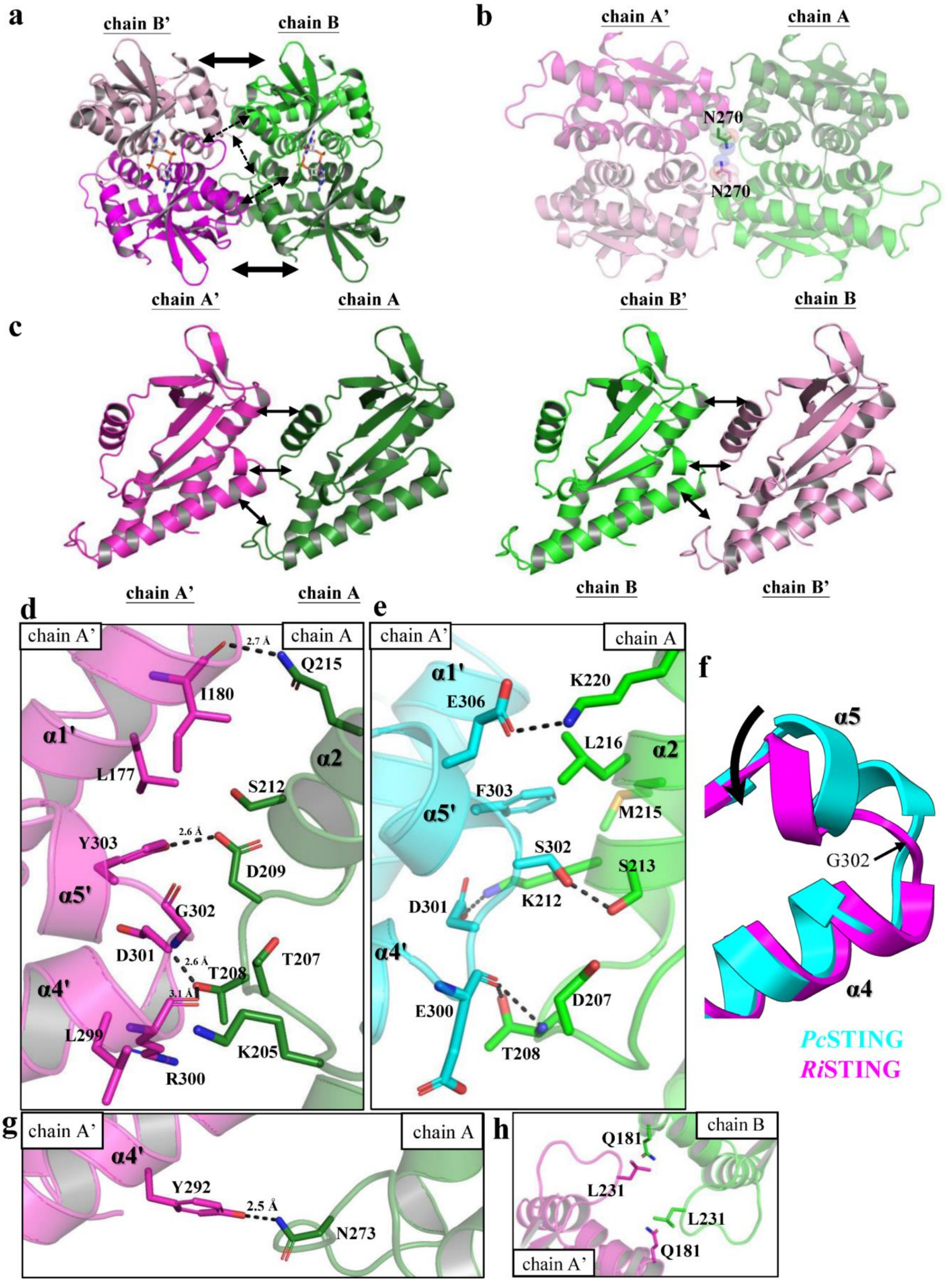

Supplementary Figure 7. *Ri*STING oligomerization revealed by crystal packing.

(a–c) Top view (a), bottom view (b), and side view (c) of the dimer (chain AB)-dimer (chain A′B′) interactions of *Ri*STING, indicated by double black arrows. In (b), the side-chain of residues N270 from both chain A′ and B interact with each other.

(d) The detailed view of the dimer-dimer interface shown in (c) between chain A and chain A' of *Ri*STING. The residues L177, I180, K205, T207, T208, D209, S212, L299, R300, D301, G302, and Y303 participate in oligomerization are indicated.

(e) The detailed view of the dimer-dimer interface between chain A and chain A' of *Pc*STING for comparison.

(f) Structural comparison of the α4-α5 loop of *Ri*STING with that of *Pc*STING. The residue G302 of *Ri*STING caused a sharp turn between α4 and α5 helices and abolished the α2-α5′ interaction in *Ri*STING in (d).

(g) The side-chain of N273 of chain A forms a H-bond (2.5 Å) with the side-chain of Y292 of chain A′ of *Ri*STING.

(h) The side-chain of L231 and Q181 form van der Waals interaction with each other from chain A′ and B. All the interacting residues are shown in sticks. The H-bonds and ionic bond are indicated in black dashed lines.

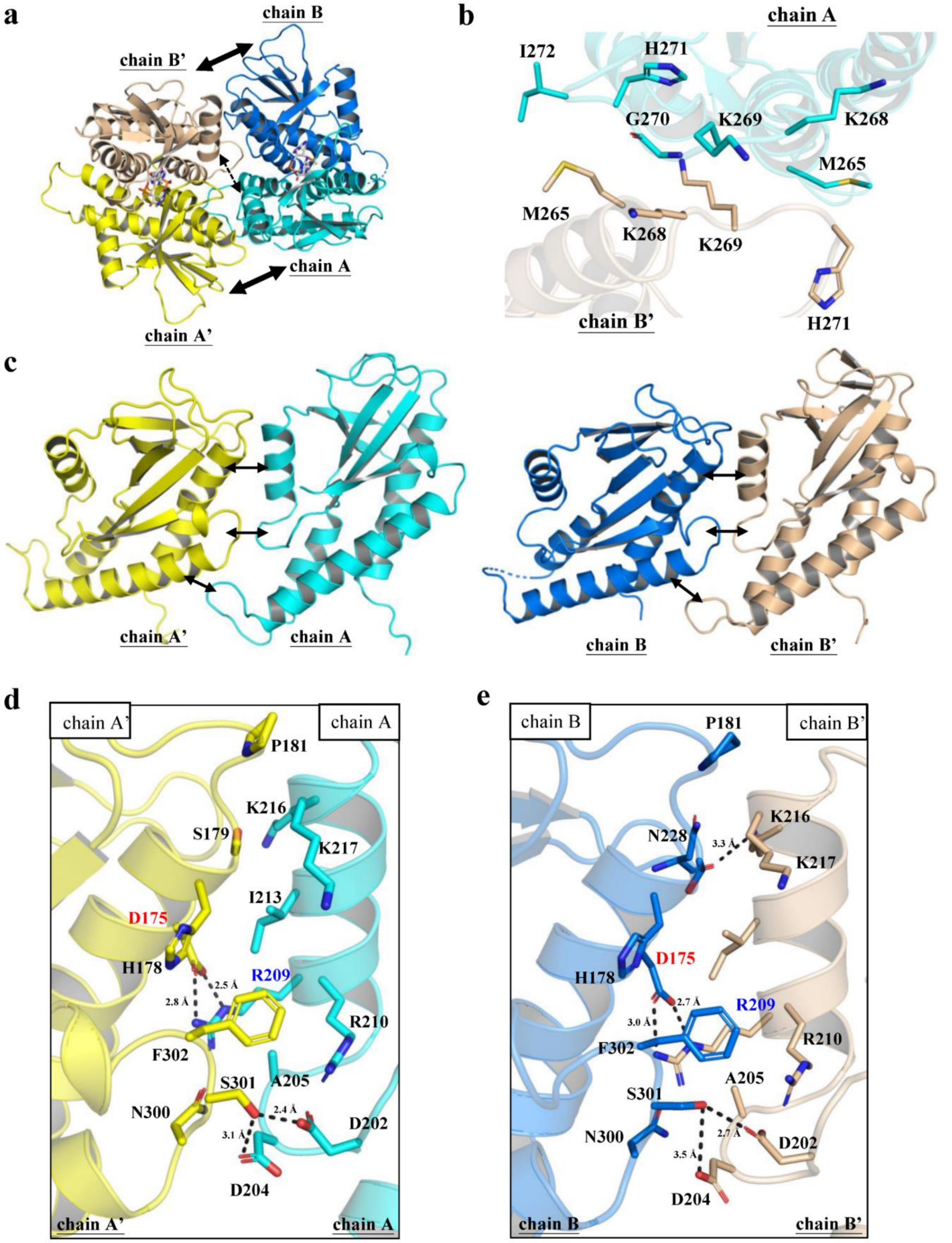

Supplementary Figure 8. *El*STING oligomerization revealed by crystal packing.

(a) Overview of the dimer (chain AB)-dimer (chain A′B′) interaction of *El*STING, indicated by double

black arrows.

(b) The detailed view of the dimer-dimer interaction between chain A and chain B' of *El*STING.

(c) The side view of the dimer-dimer interaction of *Ri*STING, indicated by double black arrows.

(d–e) The detailed view of the dimer-dimer interface shown in (c) between chain A and A' (d) and chain B and B' (e) of *El*STING. The interacting residues H178, S179, P181, A205, R209, R210, I213, K216, K217, N300, and F302 are shown in sticks and indicated. The H-bonds and ionic bonds are indicated in black dashed lines.

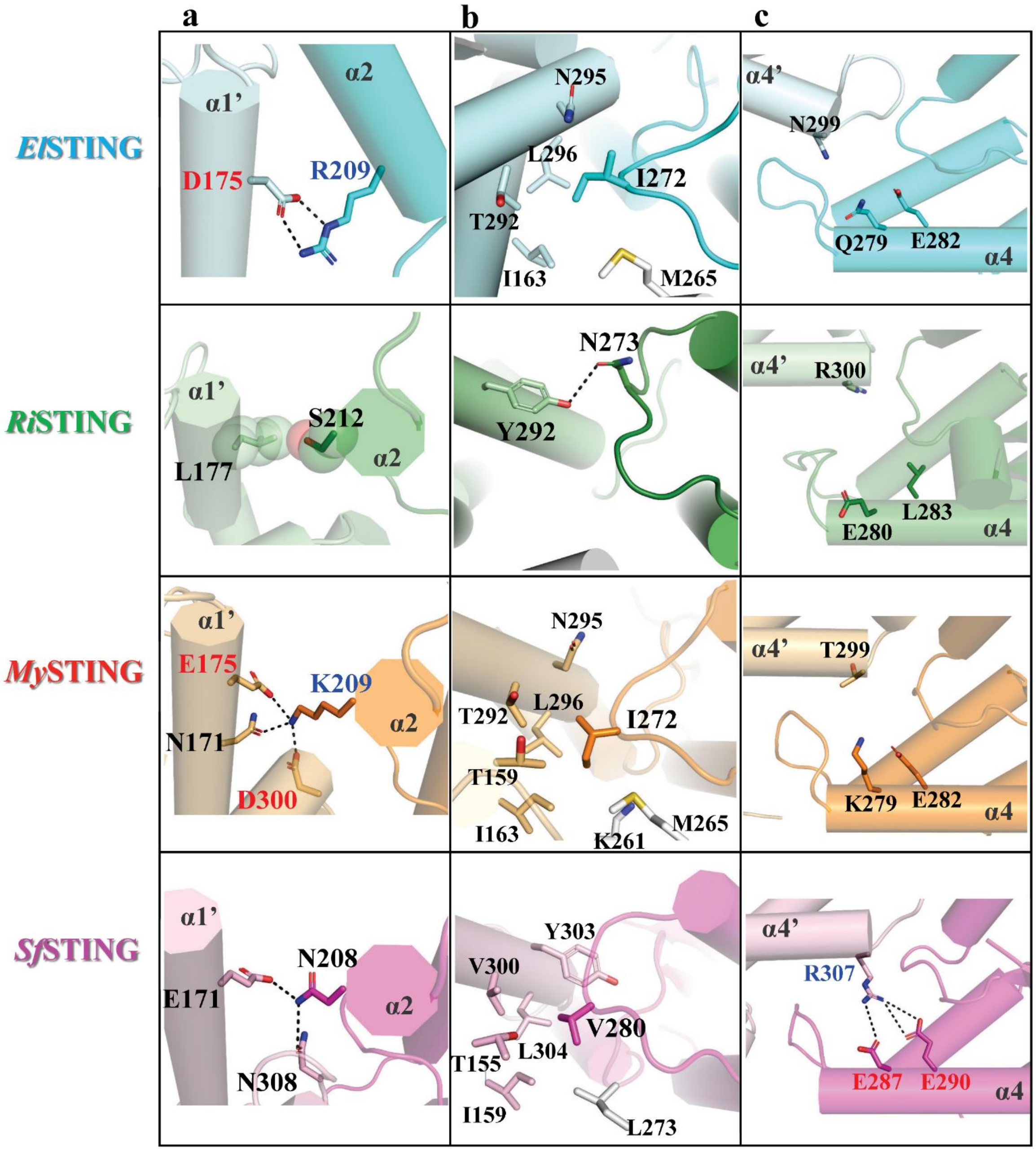

Supplementary Figure 9. Structural comparative analysis of the STING-STING contacts between *El*STING, *Ri*STING, *My*STING and *Sf*STING.

Structural comparison of (a) the interaction between α2 of one dimer and α1’ of the other dimer, (b) the contacts made by α3-α4 loop, (c) the interaction between α4 of one dimer and α4’ of the other dimer between *El*STING, *Ri*STING, *My*STING and *Sf*STING.

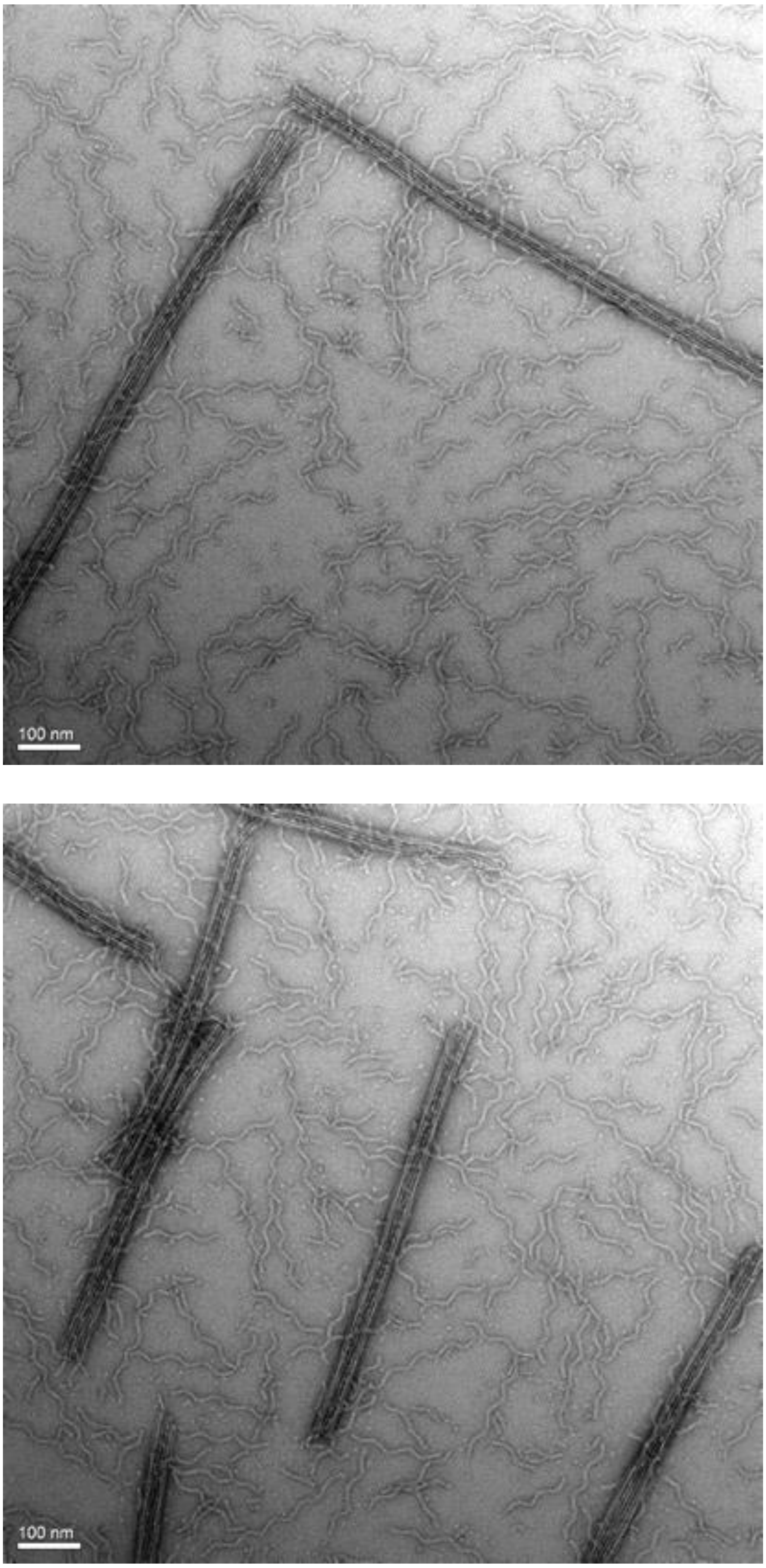

Supplementary Figure 10. The negative staining transmission electron micrographs of *El*TIR-STING captured at a magnification of 29000x.

The images are representative of $n = 9$ micrograph images.

a

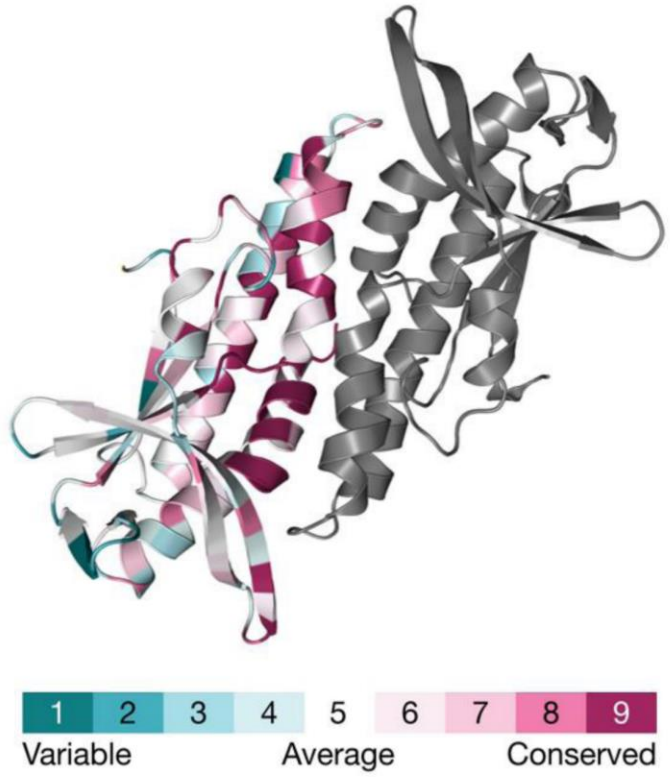

b

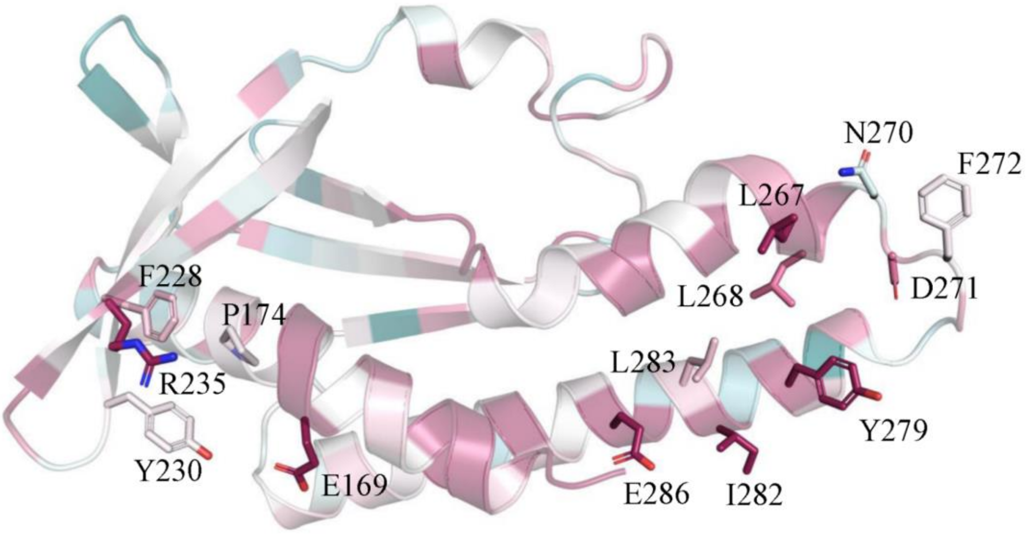

Supplementary Figure 11. Conservation analysis of residues involved in anti-parallel dimer formation of apo *Ri*STING using ConSurf webserver.

(a) The residues of one of the two protomers of anti-parallel *Ri*STING dimer are colored according to the calculated conservation scores. Conservation scores range from 1 to 9 with increasing conservation are indicated. (b) The semi-transparent cartoon model of *Ri*STING protomer colored according to conservation scores. The residues E169, F228, R235, L267, L268, D271, Y279, I282, L283, and E286 of *Ri*STING involved only in anti-parallel dimer formation, but not V-shaped dimer formation are shown in sticks and labeled.

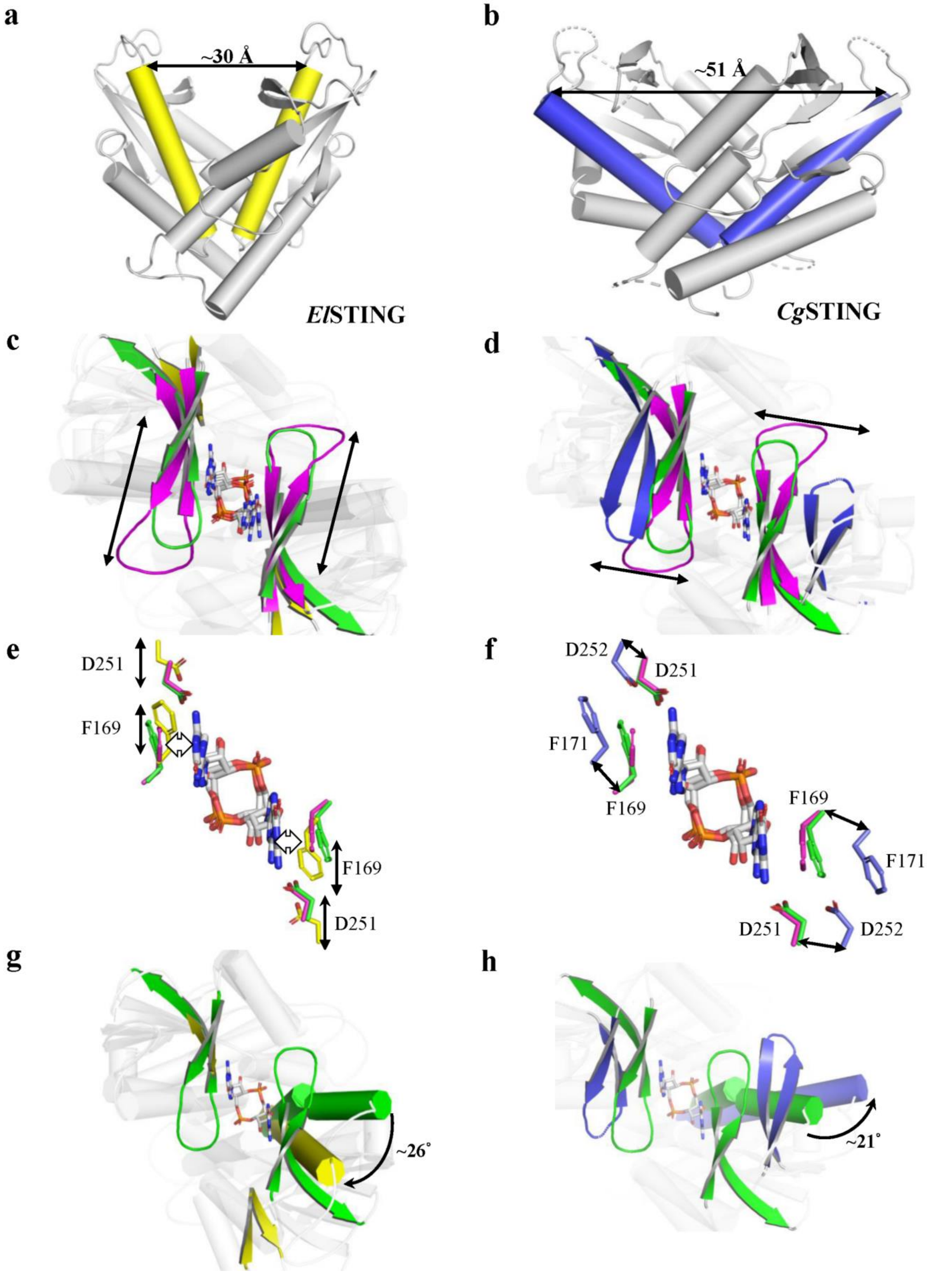

Supplementary Figure 12. Structural comparison of two ligand-free, V-shaped bacterial STING

dimers.

(a–b) The cartoon model of V-shaped dimeric architecture of (a) *El*STING (yellow) and (b) *Cg*STING (slate blue, PDB: 6WT5). The α1 helices are colored to emphasize the V-shaped architecture. The distance (Å) between two terminal ends of α1 helices are indicated.

(c–d) Superimposition of (c) *El*STING or (d) *Cg*STING with cGG-bound *Pc*STING (green, PDB: 7EBD) and cGA-bound *Fs*STING (magenta, PDB: 6WT4). The β-strand lids of them are highlighted for comparison. The movements of the β-strand lids in the presence or absence of ligand between them are indicated by arrows.

(e) The enlarged view of the ligand-binding pocket in (c).

(f) The enlarged view of the ligand-binding pocket in (d). The residues for base stacking and base recognition of *El*STING, *Cg*STING, *Pc*STING and *Fs*STING are shown in sticks.

(g–h) Superimposition of one protomer of (g) *El*STING dimer or (h) *Cg*STING dimer with one protomer of *Pc*STING dimer revealed a clockwise rotation of the other protomer of *El*STING by ~26° (g) in contrast to the anti-clockwise rotation of the other protomer of *Cg*STING by ~21° (h).

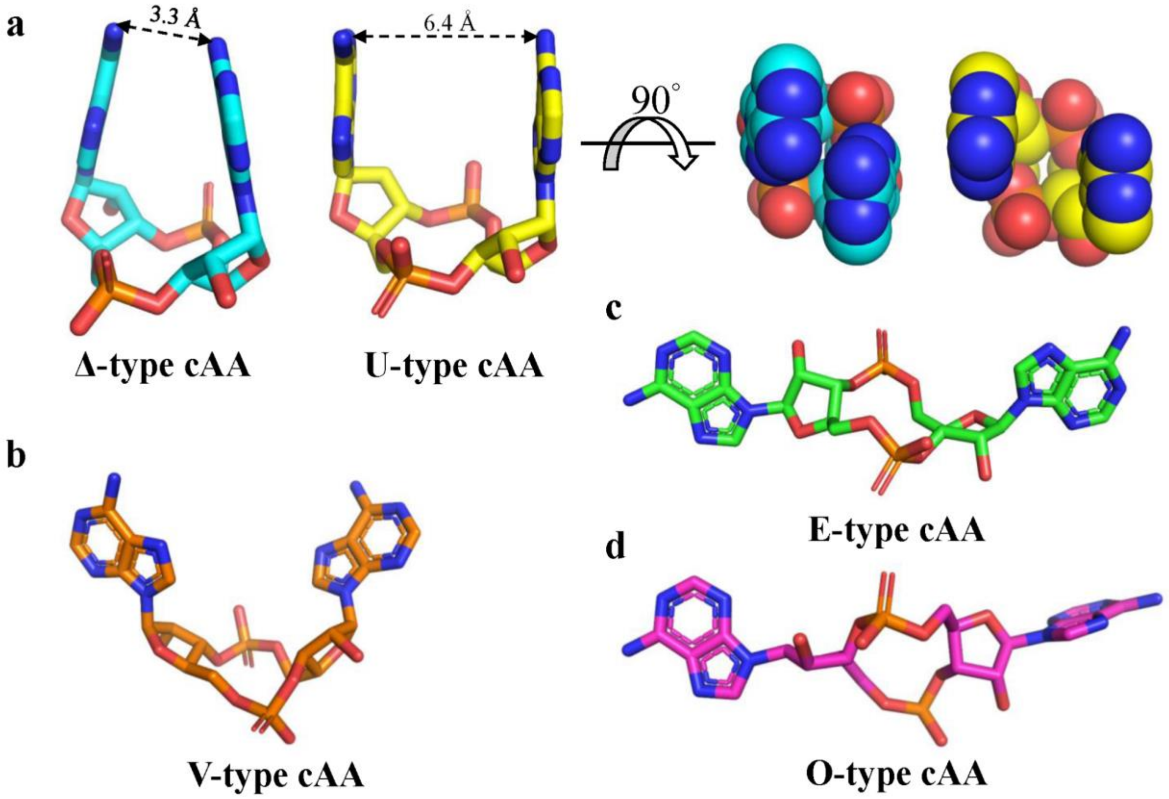

Supplementary Figure 13. Structural polymorphism of cyclic di-AMP (cAA).
(a) Comparison of the Δ-type cAA identified in the crystal structure of *El*STING_cAA with the common U-type cAA conformation. (b) V-type cAA conformation in cAA-bound phosphodiesterase PgpH HD domain from *Listeria monocytogenes* (PDB: 4S1B). (c) E-type cAA conformation in cAA-bound mouse RECON (PDB: 5UXF). (d) O-type cAA conformation in cAA-bound OpuCA CBS domain from *Listeria monocytogenes* (PDB: 5KS7).

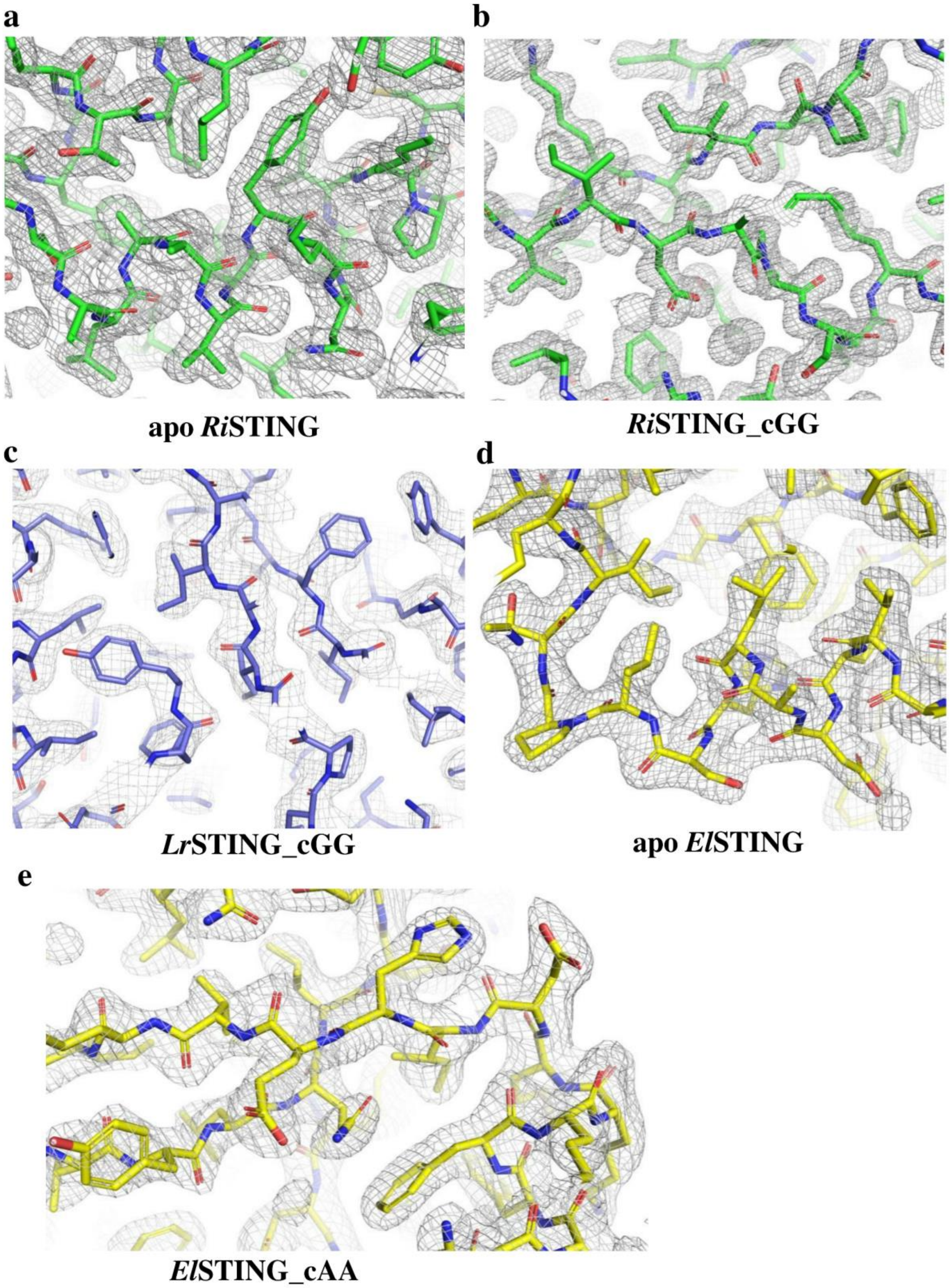

Supplementary Figure 14. Electron density maps superimposed on refined structures reported in this study.

Portion of the 2*F*o–*F*c electron-density maps of (a) apo *Ri*STING, (b) *Ri*STING_cGG, (c) *Lr*STING_cGG, (d) apo *El*STING, and (e) *El*STING_cAA contoured at the 1σ level.
