# Supplementary material for: Structural insights into the regulation, ligand recognition, and oligomerization of bacterial STING
Source: Nat Commun. 2023 Dec 21;14:8519. doi: 10.1038/s41467-023-44052-x (PMC10739871; doi:10.1038/s41467-023-44052-x)
Supplement: Supplementary file 3 — Reporting Summary [file 41467_2023_44052_MOESM3_ESM.pdf]

nature portfolio

Corresponding author(s): Yeh Chen

Last updated by author(s): Nov 8, 2023

Reporting Summary

Nature Portfolio wishes to improve the reproducibility of the work that we publish. This form provides structure for consistency and transparency in reporting. For further information on Nature Portfolio policies, see our Editorial Policies and the Editorial Policy Checklist.

Statistics

For all statistical analyses, confirm that the following items are present in the figure legend, table legend, main text, or Methods section.

n/a	Confirmed	
☐	☒	The exact sample size (*n*) for each experimental group/condition, given as a discrete number and unit of measurement
☒	☐	A statement on whether measurements were taken from distinct samples or whether the same sample was measured repeatedly
☒	☐	The statistical test(s) used AND whether they are one- or two-sided<br>*Only common tests should be described solely by name; describe more complex techniques in the Methods section.*
☒	☐	A description of all covariates tested
☒	☐	A description of any assumptions or corrections, such as tests of normality and adjustment for multiple comparisons
☐	☒	A full description of the statistical parameters including central tendency (e.g. means) or other basic estimates (e.g. regression coefficient) AND variation (e.g. standard deviation) or associated estimates of uncertainty (e.g. confidence intervals)
☒	☐	For null hypothesis testing, the test statistic (e.g. *F*, *t*, *r*) with confidence intervals, effect sizes, degrees of freedom and *P* value noted<br>*Give P values as exact values whenever suitable.*
☒	☐	For Bayesian analysis, information on the choice of priors and Markov chain Monte Carlo settings
☒	☐	For hierarchical and complex designs, identification of the appropriate level for tests and full reporting of outcomes
☒	☐	Estimates of effect sizes (e.g. Cohen's *d*, Pearson's *r*), indicating how they were calculated

Our web collection on statistics for biologists contains articles on many of the points above.

Software and code

Policy information about availability of computer code

Data collection	HKL2000_v722, TPS 13A SWAXS Data Reduction Kit (Ver. 3.6)
Data analysis	PHENIX 1.13-2998, Coot 0.9.6, PyMOL 2.3.3, NanoAnalyze v3.12.5, ATSAS 3.1.3, UCSF ChimeraX 1.5, cryoSPARC v3.3.2

For manuscripts utilizing custom algorithms or software that are central to the research but not yet described in published literature, software must be made available to editors and reviewers. We strongly encourage code deposition in a community repository (e.g. GitHub). See the Nature Portfolio guidelines for submitting code & software for further information.

Data

Policy information about availability of data

All manuscripts must include a data availability statement. This statement should provide the following information, where applicable:

- Accession codes, unique identifiers, or web links for publicly available datasets
- A description of any restrictions on data availability
- For clinical datasets or third party data, please ensure that the statement adheres to our policy

Data are available within the article and supplementary information. The coordinates and structure factors of RiSTING, RiSTING_cGG, ElSTING, ElSTING_cAA, LrSTING_cGG have been deposited in the Protein Data Bank under accession codes 8HYN [https://doi.org/10.2210/pdb8HYN/pdb], 8HY9 [https://doi.org/10.2210/pdb8HY9/pdb], 8HY8 [https://doi.org/10.2210/pdb8HY8/pdb], 8HWJ [https://doi.org/10.2210/pdb8HWJ/pdb], and 8HWI [https://doi.org/10.2210/pdb8HWI/pdb]. The protein structures used for analysis in this study are available in the Protein Data Bank under accession codes 4KSY [https://doi.org/10.2210/pdb4KSY/pdb], 5CFN [https://doi.org/10.2210/pdb5CFN/pdb], 6A03 [https://doi.org/10.2210/pdb6A03/pdb], 6Z15 [https://doi.org/10.2210/pdb6Z15/pdb], 6WT5 [https://

doi.org/10.2210/pdb6WT5/pdb], 7EBD [https://doi.org/10.2210/pdb7EBD/pdb], and 7EBL [https://doi.org/10.2210/pdb7EBL/pdb].

Human research participants

Policy information about studies involving human research participants and Sex and Gender in Research.

Reporting on sex and gender	N/A
Population characteristics	N/A
Recruitment	N/A
Ethics oversight	N/A

Note that full information on the approval of the study protocol must also be provided in the manuscript.

Field-specific reporting

Please select the one below that is the best fit for your research. If you are not sure, read the appropriate sections before making your selection.

- [x] Life sciences
- [ ] Behavioural & social sciences
- [ ] Ecological, evolutionary & environmental sciences

For a reference copy of the document with all sections, see nature.com/documents/nr-reporting-summary-flat.pdf

Life sciences study design

All studies must disclose on these points even when the disclosure is negative.

Sample size	No statistical method was used to predetermine sample size. The sample sizes were chosen based on the previously reported protocols (Ko et al. Nature Communications. 2022, 13: 26; Chang et al. Int. J. Biol. Macromol. 2023, 237: 123656).
Data exclusions	No data were excluded.
Replication	Replicates of all experiments were described in "Statistics and reproducibility" in Method section.
Randomization	For refinements of X-ray crystal structures, the 5 % randomly selected R-free reflection set was determined by an internally built algorithm of PHENIX software. For biochemical experiments, randomization was not applied, because there was no sampling requirement.
Blinding	For biochemical experiments, blinding was not applicable because researchers need to verify samples and controls for each experiment. For crystallization experiments, blinding was not applicable because the aim of the experiment is to find more crystallization hits. For in vivo experiments, although the experiments were not formally blinded, they were assay as numerically coded constructs.

Reporting for specific materials, systems and methods

We require information from authors about some types of materials, experimental systems and methods used in many studies. Here, indicate whether each material, system or method listed is relevant to your study. If you are not sure if a list item applies to your research, read the appropriate section before selecting a response.

Materials & experimental systems

n/a	Involved in the study	
x		Antibodies
x		Eukaryotic cell lines
x		Palaeontology and archaeology
x		Animals and other organisms
x		Clinical data
x		Dual use research of concern

Methods

n/a	Involved in the study	
x		ChIP-seq
x		Flow cytometry
x		MRI-based neuroimaging
